# Bilateral Renal Lymphangiectasia Associated With Polycythemia: A Rare Case Report

**DOI:** 10.7759/cureus.16130

**Published:** 2021-07-03

**Authors:** Tahereh Orouji Jokar, Ikechukwu Chidobem, Nazia Khan

**Affiliations:** 1 Hematology and Oncology, Prime Healthcare, Saint Clare's Hospital, Denville, USA; 2 Internal Medicine, Prime Healthcare, Saint Clare's Hospital, Denville, USA

**Keywords:** lymphangiectasia, renal lymphangiectasia, cystic retroperitoneal mass, perinephric lymphangiectasia, bilateral lymphangiectasia, polycythemia

## Abstract

Renal lymphangiectasia is a rare benign mesenchymal tumor of unclear etiology resulting from dilatation of perinephric lymphatic channels and formation of cystic masses. Polycythemia is a rarely associated finding with only five cases reported in the literature. We report a case of bilateral renal lymphangiectasia associated with polycythemia in a 38-year-old man who was managed conservatively with pain control. There are no clear guidelines for the management of renal lymphangiectasia; although most patients can be treated conservatively, some cases, whose diagnosis is unclear or develop complications, require definitive surgical excision.

## Introduction

Renal Lymphangiectasia, is a rare disorder of unclear etiology, with arguments in the literature both for and against an acquired versus congenital pathogenesis [1]. It is a bilateral cystic infiltration of the perirenal and parapelvic space, secondary to a miscommunication between the renal and retroperitoneal lymphatics, which results in dilatation of perinephric lymphatic channels and can lead to the formation of a unilocular or multilocular cystic mass [2]. Here, we present a case of bilateral renal lymphangiectasia associated with polycythemia.

## Case presentation

A 38-year-old male presented with right flank pain for two days. He had a past medical history significant for poorly controlled type 2 diabetes mellitus. The patient was a never-smoker and did not drink alcohol. His physical examination was positive for tenderness in the right lower abdominal quadrant and right flank, but was otherwise normal. He was not obese. Laboratory investigations revealed polycythemia (hemoglobin, hematocrit, and red blood cell count levels at 18.9 g/dl, 57.2% and 6.57 million/uL, respectively). Leukocyte count and platelet count were normal. Kidney function test was normal and urinalysis was positive for glucosuria, but was otherwise unremarkable. His polycythemia prompted molecular analysis of the patient’s blood, which was negative for Janus kinase 2 (JAK2), V617F, Exon 12, calreticulin gene (CALR), and MPL mutations, thereby making polycythemia vera an unlikely diagnosis. Erythropoietin and lactate dehydrogenase levels were also within the normal range.

**Figure 1:**
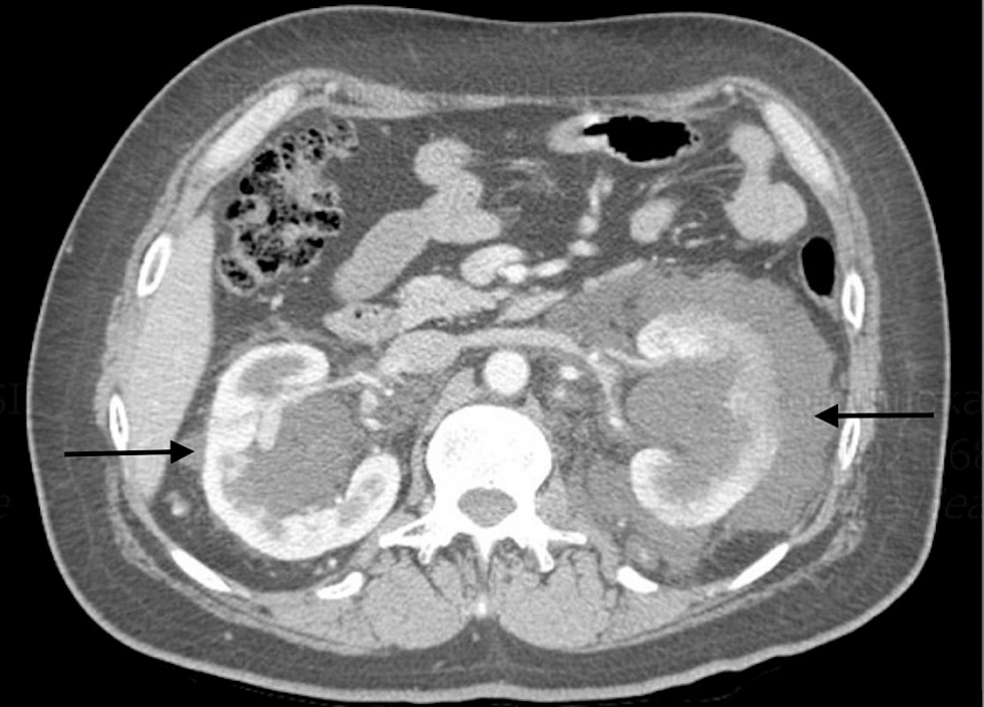
CT findings of lymphangiectasia: arrows pointing to perinephric lymphatic infiltration

**Figure 2:**
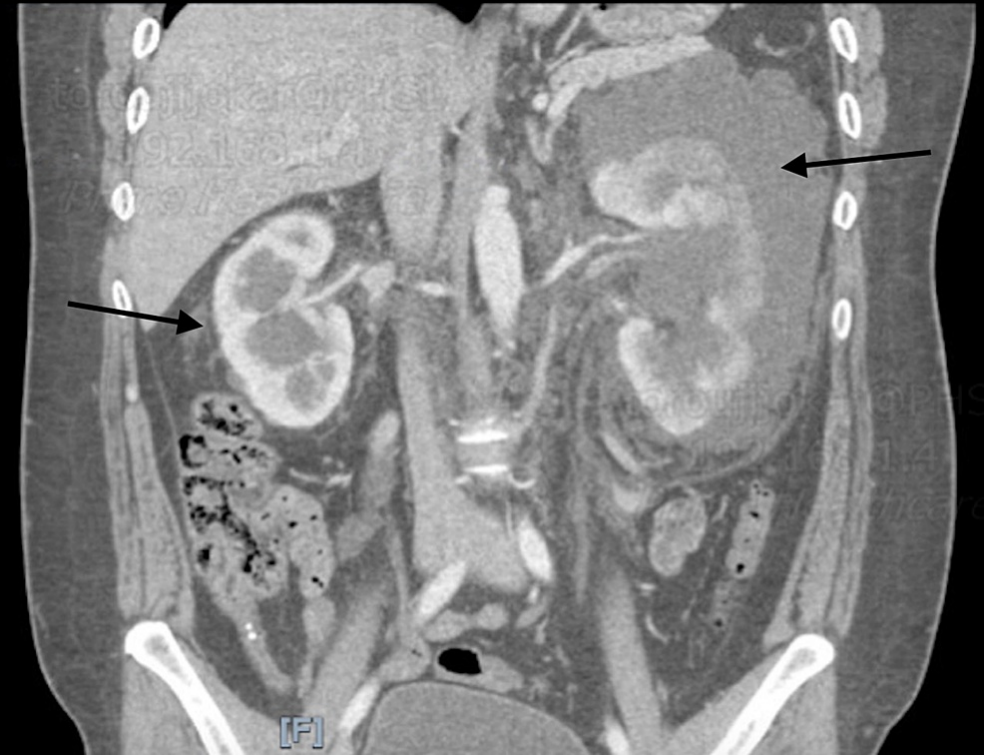
CT findings of perirenal lymphangiectasia: arrows pointing to bilateral perinephric lymphangiectasia with more pronounced changes on the left

**Figure 3:**
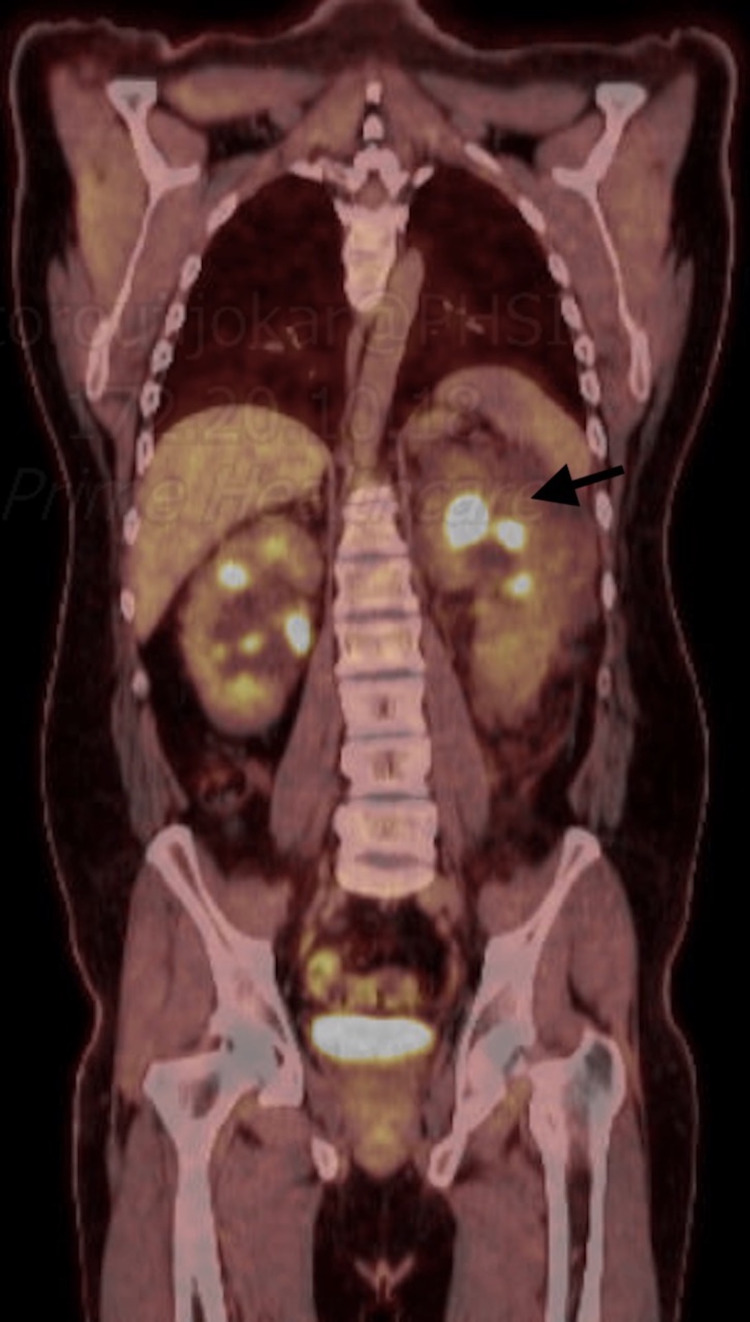
Positron emission tomography scan: arrow highlighting left perinephric infiltration with lymphatic tissue

A computed tomography (CT) scan of the abdomen/pelvis showed bilaterally enlarged kidneys with multiple bilateral parapelvic cysts, as well as infiltrating soft tissues bilaterally (left greater than the right), infiltrating nodal tissues along the roof of the mesentery extending into the right lower quadrant, and infiltrating nodal tissues within the retro-peritoneum surrounding the aorta and inferior vena cava, as well as an enhancing splenic nodule (Figures 1-2). A follow up full body fluorodeoxyglucose (FDG)-positron emission tomography (PET) scan showed a soft tissue nodule within the spleen, bilaterally enlarged kidneys with multiple bilateral parapelvic cysts, as well as bilateral pararenal hypodense soft tissues, and infiltration of soft tissues within the retroperitoneum as well as in the periaortic region (Figure 3). The patient was managed conservatively with pain control. A CT-guided aspiration and core biopsy of the left parapelvic cystic mass and left perinephric soft tissue mass was done, and the patient was discharged to outpatient follow up. The pathology report later showed no evidence of malignancy. The cystic fluid cell count and cultures were also unremarkable.

## Discussion

Renal lymphangiectasia is a rare disorder, which is usually bilateral, and unrelated to age or gender [3]. Patients with renal lymphangiectasia are usually asymptomatic but may present with abdominal distension, flank pain, abdominal pain, hypertension, hematuria, or renal vein thrombosis [1,3]. Complications described in previous reports include hemorrhage, infection, and deterioration in renal function [2,4]. Polycythemia is a very rare association of the condition, with only five cases reported in the literature [3].

The differential diagnosis of this condition includes autosomal dominant polycystic kidney disease, although in this condition the renal parenchyma is diffusely abnormal; tumors such as liposarcoma, leiomyosarcoma, fibrosarcoma, malignant teratomas, and multilocular cystic nephroma, which are predominantly cystic or necrotic, but these malignancies usually contain substantial solid components; abscess and urinoma may also produce a similar appearance, but almost all these conditions can be differentiated with the help of clinical history, normal biochemical parameters and typical imaging findings of perirenal and parapelvic involvement sparing the renal parenchyma [2,5].

The diagnosis of renal lymphangiectasia is usually made confidently on imaging (ultrasonography, CT, or MRI) due to its characteristic cystic patterns, however, as in our case, aspiration and cytological examination of the fluid may be performed if there is suspicion for malignancy [1-4,6].

There are no clear guidelines as to when conservative management, percutaneous drainage, or surgery should be used; however, treatment is generally conservative for asymptomatic patients. Complicated cases are treated with nephrectomy, percutaneous drainage, and marsupialization, however, there is a risk of an increase in the size of cysts in the contralateral kidney when a nephrectomy is performed in patients with bilateral involvement [1,3,4,5].

## Conclusions

Renal lymphangiectasia is a rare disorder, usually asymptomatic. Diagnosis is primarily radiologic, however, other modalities may be necessary to rule out confounding etiologies. Treatment varies based on symptomatic presentation and can range from observation to radical surgical resection.
